# The Effect of Disclosure on Enacted Stigma Towards Individuals with Inflammatory Bowel Disease

**DOI:** 10.1007/s10880-025-10070-8

**Published:** 2025-03-05

**Authors:** Chalé M. Jacks, Lauren A. Stutts

**Affiliations:** Davidson College, Davidson, USA

**Keywords:** Inflammatory bowel disease, Disclosure, Enacted stiga, IBD knowledge

## Abstract

Individuals with inflammatory bowel disease (IBD) often experience stigmatization. Disease disclosure has been associated with lower enacted stigma (i.e., engaging in discriminatory treatment), but minimal research has investigated IBD disclosure and enacted stigma experimentally. The present study’s aim was to examine the effect of IBD disclosure on enacted stigma in a vignette paradigm. Participants included 244 adults from the United States who were randomized into one of three vignette groups: disclosure of IBD, non-disclosure of IBD, and control (no IBD). Vignettes in both IBD groups depicted a person with frequent bowel movements. Each vignette group contained a workplace, social, and recreational setting. Participants completed measures of enacted stigma, IBD knowledge, and IBD familiarity after reading the vignettes. Participants reported greater propensity to enact stigma in response to vignettes depicting non-disclosure of IBD than vignettes depicting disclosure of IBD or no IBD and for vignettes depicting the recreational setting compared to the workplace and social setting. Enacted stigma was negatively correlated with IBD knowledge. Disease disclosure may result in decreased stigma from others. Furthermore, educating the public about IBD may be beneficial for reducing enacted stigma towards individuals with IBD.

**Supplementary Information:**

The online version contains supplementary material available at 10.1007/s10880-025-10070-8.

Inflammatory bowel disease (IBD), a chronic condition associated with the inflammation of the gastrointestinal tract, primarily consists of two diseases, Crohn’s disease and ulcerative colitis (Crohn’s & Colitis Foundation, 2024). As of 2020, approximately 2.4 million individuals in the United States (U.S.) had IBD (Lewis et al., 2023). Adults ranging from 25 to 64 years old comprise the majority of IBD cases in the U.S. (Dahlhamer, 2016). Common symptoms of IBD include abdominal pain, chronic and/or unpredictable diarrhea, vomiting, and rectal bleeding (Fakhoury et al., 2014). Furthermore, some individuals experience symptoms such as unpleasant odors, loss of bowel control, and in some cases the need for an ostomy bag, an external pouch providing an alternative route for bodily waste to leave the body (Robertson et al., 2022). However, symptoms vary in frequency, severity, and impact across individuals.

Due to IBD’s digestive symptomatology, individuals with IBD often report experiencing stigma (Taft & Keefer, 2016). In fact, Groshek et al. (2017) found that the U.S. general population had an overall negative perception of IBD and rated IBD as more stigmatizing than alcoholism, cancer, HIV/AIDS, diabetes, and obesity. One type of stigma is called enacted stigma, which refers to discriminatory behaviors toward individuals who have a certain characteristic such as a disease (Gray, 2002). Saunders (2014) found that young adults with IBD reported that they experienced enacted stigma in a variety of forms from other people including squeamish reactions, giggling about IBD, ignorance, and pity. In a systematic review, Polak et al. (2020) found that enacted stigma was experienced the most in the workplace. The stigmatization of IBD can have serious consequences (Taft et al., 2009). For example, Taft et al. (2009) found that perceived stigma is a significant predictor of poorer health-related quality of life, increased depression, increased anxiety, decreased self-esteem, and decreased medication adherence.

One way to manage stigma is through disclosure (Joachim & Acorn, 2000). Joachim and Acorn (2000) theorized that individuals engage in preventative disclosure (i.e., purposely disclose their chronic illness) with the goal of reducing others’ negative evaluations of them. Similarly, they indicated that disclosure could help the person receive sympathy and support rather than stigma. Disclosure also may be beneficial by sending the message that the condition is not something that should be shamed or hidden. That said, Guo et al. (2020) highlighted the complexity of disclosure in the IBD population as it is difficult to ascertain who to disclose to and how much to share in the disclosure. Individuals also may fear differential treatment if they disclose and/or feel that their illness experience is their private information that no one needs to know.

Disease disclosure of IBD has been examined in multiple qualitative studies (Barned et al., 2016; Frohlich, 2014; Saunders, 2014). For example, Barned et al. (2016) found that children were more likely to disclose they had IBD when they knew and trusted the person and in situations when they were asked about specific behaviors related to IBD (e.g., being absent from an event). Similarly, Saunders (2014) found that young adults tended to desire to conceal having IBD but were sometimes faced with the decision to disclose their condition when they experienced outward symptoms. They found that some of them chose to disclose, some explained their symptoms as caused by something different than IBD, and others chose not to disclose. Two common reasons when choosing not to disclose were feelings of shame and fear of stigmatization. However, Frohlich (2014) found that adults with IBD overall had positive experiences after disclosing IBD to others. It was theorized to be beneficial because they were able to educate others about their condition and feel empowered by explaining their experiences and needs.

Disease disclosure of IBD also has been examined experimentally. For example, Rohde et al. (2018) investigated how disease disclosure and severity played a role in enacted stigma towards individuals with IBD among U.S. college students. Participants were randomized to view one of six vignettes that varied with respect to the disclosure type (disclosure vs. no disclosure) and IBD disease severity (low, moderate, and high). After reading the vignette about an individual who experienced IBD symptoms during a school group project, participants answered questions about enacted stigma, their familiarity with IBD, and their perceived knowledge of IBD. They found lower levels of enacted stigma in response to the vignettes when IBD was disclosed compared to when IBD was not disclosed, which highlights the value in disclosing IBD. However, no significant interactions were found between disclosure and disease severity, suggesting the results from disclosure are independent from the severity of IBD. Additionally, they found that participants familiar with IBD displayed lower levels of enacted stigma than those unfamiliar with IBD and that perceived knowledge of IBD was negatively associated with enacted stigma. However, this study only assessed college students, did not include a control group without IBD, and only included a school-related scenario.

## Present Study

Our main aim was to examine the effect of disclosure of IBD on enacted stigma towards individuals with IBD among the general population. Our study was novel in three main ways: (1) it assessed participants from the general U.S. population, (2) it included a control group without IBD, and (3) it included three different settings for the vignettes. Participants were randomly assigned to one of three vignette groups: disclosure of IBD, non-disclosure of IBD, and control (no IBD). Each vignette group contained a workplace, social, and recreational scenario. Participants completed measures of enacted stigma, IBD knowledge, and IBD familiarity after reading the vignettes. Based on the findings from Rohde et al. (2018), we hypothesized that there would be a main effect of group such that participants would report higher enacted stigma in response to vignettes depicting non-disclosure of IBD than vignettes depicting disclosure of IBD or no IBD. In turn, we hypothesized the participants would report higher enacted stigma in response to vignettes depicting disclosure of IBD than vignettes depicting no IBD. We also hypothesized that there would be a main effect of setting such that participants would have higher enacted stigma for vignettes depicting the workplace setting than the recreational and social setting since stigma was most commonly reported in the workplace (Polak et al., 2020). Lastly, we hypothesized that enacted stigma scores would be negatively correlated with IBD knowledge and IBD familiarity, which would align with the findings from Rohde et al. (2018).

## Method

### Participants

The participants for this study met the following inclusion criteria: 18 to 99 years old, U.S. residents, and whose first language is English. Participants were recruited through Cloud Research Connect (https://www.cloudresearch.com/), an online data collection platform. Based on the results of a G*power analysis with an effect size f^2^ of 0.25, a power level of 0.90, and a significance value of 0.05, 207 participants were required for the main analysis (between-subjects ANOVA) to have sufficient statistical power (Faul et al., 2007). Participants included 267 individuals who provided consent and began the survey. However, we excluded 17 participants for non-completion (i.e., stopped participating before finishing the survey) and 6 participants for the failure to correctly answer two attention checks. Therefore, we had data for 244 participants and sufficient statistical power to conduct our analyses.

### Procedure

We secured permission from the Institutional Review Board at Davidson College prior to conducting this study, which was in accordance with the Declaration of Helsinki. After providing consent, participants were randomly assigned to one of three vignette groups, which are described in the vignettes section. Subsequently, participants completed measures of enacted stigma, IBD knowledge, IBD familiarity, and demographics. This survey took approximately 20 min to complete. Upon completion of the study, participants received $4. This study was funded by the George L. Abernethy Endowment at Davidson College.

### Vignettes

Participants were randomly assigned to one of three vignette groups: disclosure of IBD, non-disclosure of IBD, and control (no IBD) (Supplemental Information). Within each group, participants read a vignette in a workplace setting (group work project), a social setting (dinner with friends), and a recreational setting (playing tennis). Both the disclosure and non-disclosure group mentioned the hypothetical person used the restroom two or three times per hour to pass a bowel movement in that setting; however, only the disclosure group included the person disclosing their IBD diagnosis. Unlike the other experimental groups, the control group mentioned the person used the restroom once per hour and did not specify the reason as going to the bathroom once is considered normal bathroom behavior. Most people urinate on average seven to eight times per day and have a bowel movement once a day, though what is healthy and “normal” varies across individuals (Cleveland Clinic, 2023a, 2023b). Therefore, using the bathroom once during a work meeting, dinner, or recreational event would likely appear typical. These vignettes were adapted from Rohde et al.’s (2018) study who used bowel movements as their primary symptom of IBD. The symptom of bowel movements was chosen because going to the bathroom is a behavior that would be observable by others, whereas other symptoms such as abdominal pain may not be observable. Furthermore, we chose gender-neutral names and pronouns for the vignettes, so gender would not affect participants’ responses.

### Measures

#### Enacted Stigma

The Enacted Stigma Scale includes five items that assess enacted stigma towards a hypothetical person after each vignette. This measure was included in Rohde et al.’s (2018) study who modified it from Bresnahan and Zhuang (2011). The scale asked the participants whether, in response to the person depicted in the vignette, they would: (1) request to change work groups/switch friends/change partners, (2) feel embarrassed, (3) avoid the person**,** (4) feel uncomfortable being around the person, and (5) feel the person could wait to use the restroom until after the meeting. Participants rated each item on a scale ranging from 1 (*strongly disagree*) to 5 (*strongly agree*). The scale is scored by averaging the item-level ratings, with higher scores representing a greater amount of enacted stigma. In the present study, the Cronbach’s α was 0.87.

#### IBD Knowledge

The IBD Knowledge questionnaire assessed the participants' knowledge of causes, symptoms, and potential cures of IBD (Groshek et al., 2017). The questionnaire consisted of 12 true/false items (e.g., “Lack of exercise is presumed to be a leading cause of IBD”) (Groshek et al., 2017). Participants were given one point for every correct response for each item, resulting in a knowledge range for IBD from 0 to 12 with higher scores indicating higher IBD knowledge. Reliability was not assessed as each item had a correct answer.

#### IBD Familiarity

The Level of Familiarity Scale assessed each participant’s degree of contact with people living with IBD. This measure was adapted by Taft et al. (2017) from Holmes et al. (1999)’s level of contact of mental illness survey to be specific to IBD. The scale consisted of 12 items measuring the degree to which a participant has interacted with individuals known to have IBD (e.g., “I have a relative who has IBD''). While completing the scale, participants only checked off the items that applied to them. Each item had a designated score, determined by a panel of experts, signifying the level of intimacy it represented (Holmes et al., 1999). The level of intimacy ranged from 1 (*I have never observed a person that I was aware had IBD*) to 12 (*I have IBD*), with a higher level indicating a more intimate relationship with someone who has IBD. A participant’s score is based on the item with the highest level of intimacy they checked off; therefore, reliability was not assessed.

### Health and Demographic Information

If participants endorsed having IBD on the Level of Familiarity Scale, they were asked to report how long they have had the disease as well as the age they were diagnosed. Participants were also asked to report their age, gender, and race/ethnicity.

#### Attention Check

Three attention checks were included in this study. For the first attention check, there was an item following the Enacted Stigma scales asking participants to summarize the vignettes they read. The second attention check appeared at the end of the IBD Knowledge questionnaire asking participants to select “True.” For the third attention check, participants were asked to select an answer from a multiple-choice question that best described the vignettes they read at the beginning of the survey. Six participants were removed for providing an insufficient answer to the first attention check and incorrectly answering the third attention check.

### Statistical Analyses

Descriptive statistics were conducted for the main variables. We conducted chi-square analyses to determine any baseline differences among groups for gender, race, ethnicity, and IBD diagnosis. We conducted a one-way between-subjects (disclosure, non-disclosure, and control) ANOVA to assess any baseline differences in age. We conducted a 3 (disclosure, non-disclosure, and control) × 3 (workplace, social, and recreational setting) mixed ANOVA on enacted stigma. Bonferroni comparisons were used for post-hoc analyses. Pearson correlations were conducted to examine the relationships among the main variables.

## Results

### Descriptive Statistics of Participants

Descriptive statistics including gender, race, ethnicity, age, and IBD diagnosis for participants are displayed in Table 1. Most participants were White, non-Hispanic/Latinx, men. The mean age of participants was 38 years old and ranged from 18 to 72. Fifteen participants reported having IBD. The youngest was diagnosed with IBD at the age of 10, and the oldest was 50 years old. There were no significant differences by demographic variables or IBD diagnosis among the three groups (Table 1).

**Table 1:** Descriptive statistics by group

Category	Total	Disclosure Group	Non-Disclosure Group	Control Group	
	(*N* = 244)	(*n* = 85)	(*n* = 83)	(*n* = 76)	
	*n* (%)	*n* (%)	*n* (%)	*n* (%)	χ^2^
Gender					3.04
Woman	118 (48.4)	41 (48.2)	41 (49.4)	36 (47.4)	
Man	123 (50.4)	43 (50.5)	40 (48.2)	40 (52.6)	
Non-Binary	1 (0.0)	0 (0.0)	1 (1.2)	0 (0)	
Not Listed	2 (0.1)	1 (1.2)	1 (1.2)	0 (0)	
Race					18.32
White	183 (75.0)	63 (74.1)	63 (77.3)	57 (75)	
African/Am	25 (10.2)	5 (5.9)	11 (13.3)	9 (11.8)	
Asian/Am	18 (7.4)	4 (4.7)	7 (8.4)	7 (9.2)	
Bi/Multiracial	11 (4.5)	9 (10.6)	0 (0.0)	2 (2.6)	
NA/AN	3 (1.2)	1 (1.2)	1 (1.2)	1 (1.3)	
Not Listed	4 (1.6)	3 (3.5)	1 (1.2)	0 (0)	
Hispanic/Latinx	36 (14.7)	11 (12.9)	13 (15.7)	12 (15.8)	0.34
IBD Diagnosis	15 (6.1%)	5 (5.9%)	6 (7.2%)	4 (5.3%)	0.20

	*M (SD)*	*M (SD)*	*M (SD)*	*M (SD)*	*F*
Age	38.51 (11.66)	39.85 (10.98)	37.36 (11.67)	38.28 (12.36)	0.98

*African/Am* African/African American, *Asian/Am* Asian/Asian American, *NA/AN* Native American or Alaskan Native, *IBD* Inflammatory Bowel Disease

*p* > .05 for χ^2^ and *F* tests indicating no baseline differences

### Enacted Stigma by Group and Setting

**Fig. 1:**
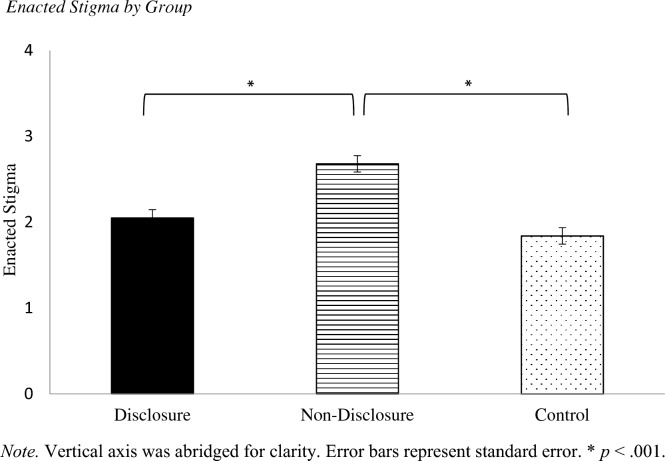
Enacted Stigma by Group. *Note.* Vertical axis was abridged for clarity. Error bars represent standard error. *p < .001

There was not a significant interaction between group and setting, *F*(4, 482) = 2.38, *p* = 0.051, η_p_^2^ = 0.019. However, there was a main effect of group, *F*(2, 241) = 20.35,* p* < 0.001, η_p_^2^ = 0.14 (Fig. 1). Post hoc results revealed that participants reported higher enacted stigma in response to vignettes depicting non-disclosure of IBD (*M* = 2.68, *SD* = 0.99) than vignettes depicting disclosure of IBD (*M* = 2.05, *SD* = 0.87) or no IBD/control (*M* = 1.84, *SD* = 0.74), *p* < 0.001 for both. However, there was no significant difference in enacted stigma in response to vignettes depicting disclosure of IBD and no IBD/control, *p* = 0.36.

**Fig. 2:**
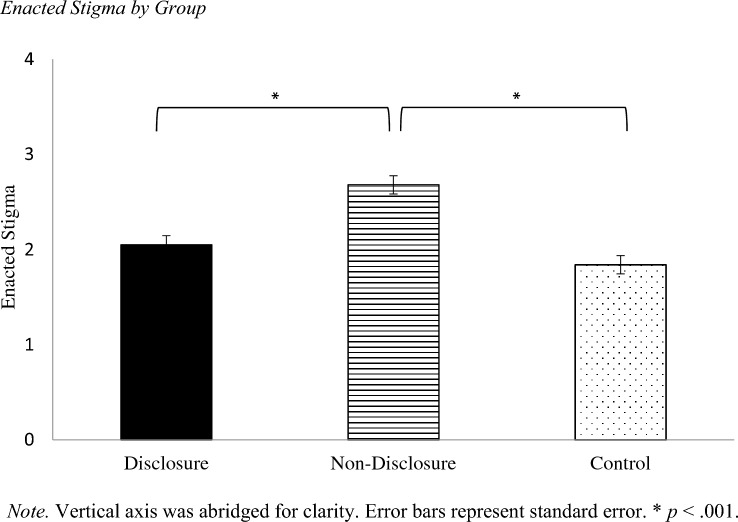
Enacted Stigma by Setting. *Note.* Vertical axis was abridged for clarity. Error bars represent standard error. *p < .001

There also was a main effect of setting, *F*(2, 482) = 19.16,* p* < 0.001, η_p_^2^ = 0.074 (Fig. 2). Post hoc results revealed that participants reported higher enacted stigma for vignettes depicting the recreational setting (*M* = 2.31, *SD* = 1.04) than the workplace setting (*M* = 2.17, *SD* = 0.93) and social setting (*M* = 2.10, *SD* = 1.01), *p* < 0.001 for both. There was no significant difference in enacted stigma between the workplace setting and social setting, *p* = 0.10.

Table 2 contains the descriptive statistics of each group by individual enacted stigma item across all settings. For the disclosure group, mean item scores were highest for “want to change groups” and “feel embarrassed” and the lowest for “feel the person could wait to use the restroom.” For the non-disclosure and control group, mean item scores were highest for “want to change groups” and “feel the person could wait to use the restroom” and the lowest for “avoid the person.”

**Table 2:** Descriptive statistics by group by each enacted stigma item averaged across settings

Item	Total	Disclosure Group	Non-Disclosure Group	Control Group
	(*N* = 244)	(*n* = 85)	(*n* = 83)	(*n* = 76)
	*M* (*SD*)	*M* (*SD*)	*M* (*SD*)	*M* (*SD*)
Want to change groups	2.47 (1.07)	2.33 (1.01)	3.00 (1.04)	2.03 (0.91)
Feel embarrassed	2.11 (1.11)	2.17 (1.15)	2.51 (1.19)	1.61 (0.74)
Avoid the person	1.97 (0.96)	1.90 (0.95)	2.42 (1.54)	1.54 (0.65)
Feel uncomfortable	2. 10 (1.10)	2.07 (1.10)	2.55 (1.21)	1.65 (0.76)
Feel the person could wait to use the restroom	2.35 (1.20)	1.79 (0.92)	2.94 (1.23)	2.34 (1.15)

### Descriptive Statistics and Correlations

IBD knowledge was relatively low; participants on average correctly answered 7 out of 12 items (*M* = 7.30, *SD* = 1.78), which is approximately 58% correct. Similarly, IBD familiarity was low (*M* = 5.72, *SD* = 3.88) as 12 indicated the highest familiarity. Higher enacted stigma was associated with lower IBD knowledge, *r* = -0.23, *p* < 0.001. However, enacted stigma was not significantly associated with IBD familiarity, *r* = -0.03, *p* = 0.70, and IBD knowledge was not associated with IBD familiarity, *r* = -0.12, *p* = 0.08.

## Discussion

Our aim was to examine the effect of disclosure of IBD on enacted stigma towards individuals with IBD among the general population. Our hypotheses were partially supported. Our hypothesis that participants would report higher enacted stigma in response to vignettes depicting non-disclosure of IBD than vignettes depicting disclosure of IBD or no IBD was supported. However, there was not a significant difference in enacted stigma in response to vignettes depicting disclosure of IBD and no IBD, which was unexpected. We also were surprised that participants reported higher enacted stigma for vignettes depicting the recreational setting than the workplace setting and social setting. Consistent with our hypothesis, we found that enacted stigma was negatively correlated with IBD knowledge; however, enacted stigma was not associated with IBD familiarity.

As expected, participants reported significantly lower enacted stigma in response to vignettes depicting disclosure of IBD than those depicting non-disclosure of IBD. This result aligns with Rohde et al.’s (2018) study that found lower levels of enacted stigma in groups where disclosure was made. This result can be explained through Joachim and Acorn’s (2000) theory that preventative disclosure reduces negative evaluations. When looking at our item-level responses, it appears that disclosure produced an increased understanding of IBD. For example, the lowest level of stigma for the disclosure group was the item that they felt the person could wait to use the restroom, whereas that item was one of the highest levels of stigma for the non-disclosure group and control group. Therefore, it appears that learning the person had IBD was helpful in improving participants’ understanding of why they may not be able to wait to use the restroom.

We also found that participants reported higher enacted stigma in response to vignettes depicting non-disclosure of IBD than the vignettes depicting no IBD. Higher levels of enacted stigma in the non-disclosure group can most likely be attributed to the participant’s lack of understanding regarding the atypical bowel movements of the person in the vignette (Rohde et al., 2018). Because the non-disclosure vignette did not provide an explanation for the frequent bowel movements, this likely made it more difficult for participants to sympathize with the person in the vignette. The lower levels of enacted stigma in the control group most likely stems from the portrayal of normal bathroom behavior (Cleveland Clinic, 2023a, 2023b).

However, we did not find a difference in enacted stigma in response to vignettes depicting disclosure of IBD and the vignettes depicting no IBD, which was unexpected. The similarity between these groups in conjunction with the significant differences between the non-disclosure and control group suggest it may not be the behavior (i.e., using the restroom two or three times per hour) that people are discriminating against, but the lack of understanding that the person has a chronic illness. This is in concert with Rohde et al.’s (2018) finding that the disclosure of IBD did not affect stigma differently at low, medium, and high levels of severity. This also highlights the value of disclosure as the individual who had abnormal bathroom behavior and explained it was treated similarly as the person who had normal bathroom behavior.

Surprisingly, we found that participants reported higher enacted stigma for vignettes depicting the recreational setting than the workplace setting and social setting. This finding contradicts Polak et al.’s (2020) study, which highlighted that enacted stigma was most prevalent in the workplace. The higher level of enacted stigma from the recreational setting for the present study may stem from the type of recreational activity chosen. Tennis requires all players be present in order for the match to occur. Therefore, if someone is needing to leave the match, then it will most likely cause a disruption to the game. In contrast, leaving a meeting at work or in a social dinner setting may not have as strong of consequences as they may be less needed for those contexts to function. Furthermore, bathroom access may have been perceived as easier in a workplace or social setting and may have been perceived as less convenient in a recreational setting.

As expected, enacted stigma was negatively correlated with IBD knowledge, which aligns with previous research (Rohde et al., 2018). One possible explanation could be that people who are more knowledgeable are also more sympathetic. If someone knows more about the symptomatology of IBD, then they may be more understanding when someone with the disease has to repeatedly use the restroom in a short period of time. However, IBD knowledge was low in our sample as most participants only knew the correct answer for around half the responses. This result suggests the need for educational campaigns to increase IBD knowledge.

In contrast to our hypothesis, enacted stigma was not significantly correlated with IBD familiarity. This result is surprising as previous research found that higher IBD-related stigma was associated with lower familiarity with IBD (Taft et al., 2017). One reason for this difference in results between our study and Taft et al.’s (2017) study is that we used different measures for stigma. For example, our study only assessed enacted stigma, whereas Taft et al.’s (2017) study examined IBD stigma including enacted and internalized stigma. It is possible that only some forms of stigma are associated with disease familiarity. Future research should explore this relationship further.

One limitation of this study was that it was composed mainly of White and non-Hispanic participants, which reduces its generalizability to a more diverse population. In addition, we only used one example for each type of setting (workplace, social, and recreational). Other examples from those settings (e.g., going to a concert, playing golf) could have produced different results. We also only included one IBD symptom of going to the bathroom. Other symptoms such as having an ostomy bag may have differentially affected stigma. Moreover, due to the self-report nature of the study, participants may have under-reported their level of enacted stigma to appear less judgmental. We also did not have baseline scores of enacted stigma toward IBD; therefore, we could not examine change in stigma over time within participants. Furthermore, we only have correlations among our variables and are unable to make claims about causality among variables.

Despite those limitations, the main implication of our study is that disclosure is beneficial in reducing enacted stigma toward individuals with IBD. Healthcare professionals could advise their patients with IBD to disclose their disease in situations where they feel it would benefit others to understand their bathroom habits. Individuals with IBD may benefit from disclosure in a variety of contexts such as work, social, and recreational settings. However, individuals may experience difficulties with disclosure in not knowing what to say and how many details to include. Similar to previous research, telling trusted individuals is usually a safe place to start with disclosure (Barned et al., 2016). Individuals may also benefit from having a plan on how to respond to various reactions they might receive. Peer support for managing the challenges of disease disclosure would likely be valuable. That said, it is also important to acknowledge that disclosure may not be best for everyone and for every situation. Individuals with IBD need to evaluate their own feelings, specific situation, the people involved, and the potential advantages and disadvantages of disclosing to decide what works best for them.

In addition to disclosure, education may play an important role in decreasing the level of enacted stigma towards individuals with IBD from the general population. Our study demonstrated that the majority of participants had low levels of IBD knowledge. Therefore, it would benefit individuals with IBD if the general population was more educated about the disease. The Crohn’s and Colitis Foundation is a valuable way for individuals to learn more about IBD as they have free fact sheets, education programs, and informative blog posts (https://www.crohnscolitisfoundation.org/). Another possible way to educate the general population is through social media and advertising. For example, Frohlich (2016) highlighted the value of making IBD more visible through social media channels. For example, individuals with IBD could post photos of scars or ostomies, share one’s experiences and stories, promote fundraising organizations for IBD, and provide education about IBD online. There are many informative posts on Instagram under the hashtags of #inflammatory bowel disease, #crohnsdisease, #colitis, and #ibdvisible, among others. Moreover, education about medical diseases could be more integrated into the public school system.

This study highlights the value of disease disclosure in reducing enacted stigma of IBD. Future studies could implement a variety of symptoms in the vignettes to see whether certain symptoms play a role on enacted stigma levels. Furthermore, future research efforts should study the effect of disclosure on enacted stigma among a more diverse population. Ultimately, continuing this research will inform future interventions for individuals with IBD regarding disease disclosure.

## Supplementary Information

Below is the link to the electronic supplementary material.

Supplementary file1 (DOCX 17 KB)

## Data Availability

Data may be made available by request of the corresponding author.
