# Supplementary material for: The Effect of Disclosure on Enacted Stigma Towards Individuals with Inflammatory Bowel Disease
Source: J Clin Psychol Med Settings. 2025 Mar 5;32(3):517–25. doi: 10.1007/s10880-025-10070-8 (PMC12370857; doi:10.1007/s10880-025-10070-8)
Supplement: Supplementary file 1 — Supplementary file1 (DOCX 17 KB) [file 10880_2025_10070_MOESM1_ESM.docx]

**Supplemental Information**

Vignette Introduction:

You will first read and rate three vignettes. After those three vignettes, you will then be asked to provide a brief written summary of the theme of the vignettes after reading all of them. Therefore, please read the vignettes carefully.

Disclosure Group Vignettes:

Workplace Vignette

- Every Monday, you meet with several of your colleagues to work on a project. One of your coworkers, Sam, discloses that they have inflammatory bowel disease, which they say causes them to need to use the bathroom frequently. As a result, Sam generally uses the restroom two or three times per hour to pass a bowel movement during group meetings.

Based on this scenario I would…

1) request to change work groups

1-strongly disagree, 2-disagree, 3-neutral, 4-agree, 5- strongly agree

2) feel embarrassed

1-strongly disagree, 2-disagree, 3-neutral, 4-agree, 5- strongly agree

3) avoid Sam

1-strongly disagree, 2-disagree, 3-neutral, 4-agree, 5- strongly agree

4) feel uncomfortable being around Sam

1-strongly disagree, 2-disagree, 3-neutral, 4-agree, 5- strongly agree

5) feel Sam could wait to use the restroom until after the meeting

1-strongly disagree, 2-disagree, 3-neutral, 4-agree, 5- strongly agree

Social Vignette

- Every Wednesday, you meet with several of your friends for dinner. One of your friends, Alex, discloses that they have inflammatory bowel disease, which they say causes them to need to use the bathroom frequently. As a result, Alex generally uses the restroom two or three times per hour to pass a bowel movement during dinner.

Based on this scenario I would…

1) want to switch friends

1-strongly disagree, 2-disagree, 3-neutral, 4-agree, 5- strongly agree

2) feel embarrassed

1-strongly disagree, 2-disagree, 3-neutral, 4-agree, 5- strongly agree

3) avoid Alex

1-strongly disagree, 2-disagree, 3-neutral, 4-agree, 5- strongly agree

4) feel uncomfortable being around Alex

1-strongly disagree, 2-disagree, 3-neutral, 4-agree, 5- strongly agree

5) feel Alex could wait to use the restroom until after dinner was over

1-strongly disagree, 2-disagree, 3-neutral, 4-agree, 5- strongly agree

Recreation Vignette

- Every Friday, you meet with several of your friends to play tennis. One of your friends, Taylor, discloses that they have inflammatory bowel disease, which they say causes them to need to use the bathroom frequently. As a result, Taylor generally uses the restroom two or three times per hour to pass a bowel movement during a match.

Based on this scenario I would…

1) request to change partners

1-strongly disagree, 2-disagree, 3-neutral, 4-agree, 5- strongly agree

2) feel embarrassed

1-strongly disagree, 2-disagree, 3-neutral, 4-agree, 5- strongly agree

3) avoid Taylor

1-strongly disagree, 2-disagree, 3-neutral, 4-agree, 5- strongly agree

4) feel uncomfortable being around Taylor

1-strongly disagree, 2-disagree, 3-neutral, 4-agree, 5- strongly agree

5) feel Taylor could wait to use the restroom until after the match

1-strongly disagree, 2-disagree, 3-neutral, 4-agree, 5- strongly agree

Non-disclosure Group Vignettes:

Workplace Vignette

- Every Monday, you meet with several of your coworkers to work on a project. One of your coworkers, Sam, generally uses the restroom two or three times per hour to pass a bowel movement during group meetings.

Based on this scenario I would…

1) request to change work groups

1-strongly disagree, 2-disagree, 3-neutral, 4-agree, 5- strongly agree

2) feel embarrassed

1-strongly disagree, 2-disagree, 3-neutral, 4-agree, 5- strongly agree

3) avoid Sam

1-strongly disagree, 2-disagree, 3-neutral, 4-agree, 5- strongly agree

4) feel uncomfortable being around Sam

1-strongly disagree, 2-disagree, 3-neutral, 4-agree, 5- strongly agree

5) feel Sam could wait to use the restroom until after the meeting

1-strongly disagree, 2-disagree, 3-neutral, 4-agree, 5- strongly agree

Social Vignette

- Every Wednesday, you meet with several of your friends for dinner. One of your friends, Alex, generally uses the restroom two or three times per hour to pass a bowel movement during dinner.

Based on this scenario I would…

1) want to switch friends

1-strongly disagree, 2-disagree, 3-neutral, 4-agree, 5- strongly agree

2) feel embarrassed

1-strongly disagree, 2-disagree, 3-neutral, 4-agree, 5- strongly agree

3) avoid Alex

1-strongly disagree, 2-disagree, 3-neutral, 4-agree, 5- strongly agree

4) feel uncomfortable being around Alex

1-strongly disagree, 2-disagree, 3-neutral, 4-agree, 5- strongly agree

5) feel Alex could wait to use the restroom until after dinner was over

1-strongly disagree, 2-disagree, 3-neutral, 4-agree, 5- strongly agree

Recreation Vignette

- Every Friday, you meet with several of your friends to play tennis. One of your friends, Taylor, generally uses the restroom two or three times per hour to pass a bowel movement during a match.

Based on this scenario I would…

1) request to change partners

1-strongly disagree, 2-disagree, 3-neutral, 4-agree, 5- strongly agree

2) feel embarrassed

1-strongly disagree, 2-disagree, 3-neutral, 4-agree, 5- strongly agree

3) avoid Taylor

1-strongly disagree, 2-disagree, 3-neutral, 4-agree, 5- strongly agree

4) feel uncomfortable being around Taylor

1-strongly disagree, 2-disagree, 3-neutral, 4-agree, 5- strongly agree

5) feel Taylor could wait to use the restroom until after the match

1-strongly disagree, 2-disagree, 3-neutral, 4-agree, 5- strongly agree

No IBD/Control Group Vignettes:

Workplace Vignette

- Every Monday, you meet with several of your coworkers to work on a project. One of your coworkers, Sam, generally uses the restroom once per hour during group meetings.

Based on this scenario I would…

1) request to change work groups

1-strongly disagree, 2-disagree, 3-neutral, 4-agree, 5- strongly agree

2) feel embarrassed

1-strongly disagree, 2-disagree, 3-neutral, 4-agree, 5- strongly agree

3) avoid Sam

1-strongly disagree, 2-disagree, 3-neutral, 4-agree, 5- strongly agree

4) feel uncomfortable being around Sam

1-strongly disagree, 2-disagree, 3-neutral, 4-agree, 5- strongly agree

5) feel Sam could wait to use the restroom until after the meeting

1-strongly disagree, 2-disagree, 3-neutral, 4-agree, 5- strongly agree

Social Vignette

- Every Wednesday, you meet with several of your friends for dinner. One of your friends, Alex, generally uses the restroom once per hour during dinner.

Based on this scenario I would…

1) want to switch friends

1-strongly disagree, 2-disagree, 3-neutral, 4-agree, 5- strongly agree

2) feel embarrassed

1-strongly disagree, 2-disagree, 3-neutral, 4-agree, 5- strongly agree

3) avoid Alex

1-strongly disagree, 2-disagree, 3-neutral, 4-agree, 5- strongly agree

4) feel uncomfortable being around Alex

1-strongly disagree, 2-disagree, 3-neutral, 4-agree, 5- strongly agree

5) feel Alex could wait to use the restroom until after dinner was over

1-strongly disagree, 2-disagree, 3-neutral, 4-agree, 5- strongly agree

Recreation Vignette

- Every Friday, you meet with several of your friends to play tennis. One of your friends, Taylor, generally uses the restroom once per hour during a match.

Based on this scenario I would…

1) request to change partners

1-strongly disagree, 2-disagree, 3-neutral, 4-agree, 5- strongly agree

2) feel embarrassed

1-strongly disagree, 2-disagree, 3-neutral, 4-agree, 5- strongly agree

3) avoid Taylor

1-strongly disagree, 2-disagree, 3-neutral, 4-agree, 5- strongly agree

4) feel uncomfortable being around Taylor

1-strongly disagree, 2-disagree, 3-neutral, 4-agree, 5- strongly agree

5) feel Taylor could wait to use the restroom until after the match

1-strongly disagree, 2-disagree, 3-neutral, 4-agree, 5- strongly agree

**Summary**

Please briefly summarize the main content of the vignette (e.g., what was a common theme across the vignettes?)
